# Enhancing Mechanical and Biodegradation Properties of Zn-0.5Fe Alloys Through Rotary Forging

**DOI:** 10.3390/ma18030722

**Published:** 2025-02-06

**Authors:** Lebin Tang, Hailing Chen, Xinglong Zhu, Muhammad Zubair, Tao Sun, Lijing Yang, Xiang Lu, Zhenlun Song

**Affiliations:** 1 State Key Laboratory of Advanced Marine Materials, Ningbo Institute of Materials Technology and Engineering, Chinese Academy of Sciences, Ningbo 315201, China; tanglebin@nimte.ac.cn (L.T.); chenhailing@nimte.ac.cn (H.C.); zhuxinglong@nimte.ac.cn (X.Z.); zubairsharif323@outlook.com (M.Z.); suntao@nimte.ac.cn (T.S.); luxiang@nimte.ac.cn (X.L.); songzhenlun@nimte.ac.cn (Z.S.); 2 University of Chinese Academy of Sciences, Beijing 100049, China

**Keywords:** Zn-Fe alloy, rotary forging, grain refinement, mechanical properties, biodegradable metals

## Abstract

The rising prevalence of orthopedic conditions, driven by an aging population, has led to a growing demand for advanced implant materials. Traditional metals such as stainless steel and titanium alloys are biologically inert and often necessitate secondary surgical removal, imposing both economic and psychological burdens on patients. Biodegradable zinc-based alloys offer promising alternatives due to their moderate degradation rates, biocompatibility, and tissue-healing properties. However, existing studies on Zn-Fe alloys primarily focus on composition optimization, with limited investigation into how processing methods influence their performance. This study explores the effects of rotary forging on the microstructure and mechanical properties of Zn-0.5Fe alloys. By refining grain structure and promoting dynamic recrystallization, rotary forging achieves significant improvements in ductility (60% elongation, a 114% increase compared to the extruded state) while maintaining corrosion resistance. Electrochemical and immersion tests reveal that rotary forging produces a denser and more protective corrosion layer, thereby improving the degradation performance of the material in simulated body fluid. Cytotoxicity and fluorescence staining tests confirm excellent biocompatibility, validating the material’s suitability for medical applications. These findings elucidate the mechanisms by which rotary forging enhances the properties of Zn-0.5Fe alloys, providing a novel approach to tailoring biodegradable implant materials for orthopedic applications.

## 1. Introduction

In recent years, orthopedic morbidity has risen significantly, driven by the rapid growth of the aging population. Traditional bone implants, fabricated from medical-grade metals such as stainless steel, titanium (Ti) alloys, and cobalt–nickel alloys, are biologically inert upon implantation and typically require removal through a secondary surgery at the end of their service life. This necessitates additional economic costs for patients and adversely affects their physical and mental health [1]. Biodegradable metallic materials represent a category of biomaterials that can undergo gradual degradation in body fluids. The corrosion products produced during this process can promote tissue healing, effectively eliminating the need for secondary surgical removal [2]. At present, biodegradable metals are primarily classified into three categories: ferric (Fe) alloys, magnesium (Mg) alloys, and zinc (Zn) alloys. Among these, Mg alloys have been the focus of early research on biodegradable metals. However, Mg alloys corrode too quickly during the degradation process, making it difficult to meet the long-term service requirements of implants. Additionally, hydrogen gas is produced during degradation, which can lead to subcutaneous emphysema [3]. The use of Fe alloys in bone implant materials is constrained by their slow degradation rates and incomplete degradation upon reaching the end of their service life [4]. In recent years, Zn and Zn alloys have become research hotspots for biodegradable metals. As one of the trace elements in the human body, Zn plays a crucial role in various biological functions, such as signaling, gene expression, and nucleic acid metabolism, and has good biocompatibility [5]. In addition, the standard electrode potential of Zn is between that of Fe and Mg, and the degradation rate is moderate. Therefore, degradable Zn alloys have significant potential for application in orthopedics [6]. Surface treatments and coatings have also emerged as promising strategies to enhance the performance of zinc-based biomedical implants. For instance, phosphate-based coatings have been shown to improve the corrosion resistance of Zn in physiological environments [7], while multifunctional surface treatments can provide enhanced biocompatibility and controlled degradation behavior [8]. Combining such strategies with processing techniques could further optimize the properties of Zn alloys for clinical applications.

Zn-Fe alloys could be employed as a prospective metallic material for medical applications. Both Zn and Fe are essential elements that are naturally metabolized and released in the body, contributing to the restoration of injured tissue [9]. Moreover, Fe is considered a suitable alloying element for Zn [10,11]. Su et al. [12] incorporated Fe into Zn and demonstrated that the uniformly distributed second phase significantly improved the mechanical properties of the alloys. This addition also promoted uniform degradation, thereby improving the biocompatibility of the alloys, particularly in the case of Zn-0.4Fe alloys. Králová et al. [10] conducted a systematic study to investigate the effect of different Fe contents (0–10 wt%) on the mechanical, corrosion, and biological properties of Zn-xFe alloys. The results showed that the addition of Fe up to 5 wt% is an effective approach for modifying Zn-based materials to accelerate the degradation process. However, due to the hexagonal close-packed structure of Zn, Zn-Fe alloys exhibit poor plasticity at room temperature [13]. Shi et al. [14] developed a bottom-cycle water-cooled casting method to produce Zn-0.3Fe alloys, which were significantly refined by the FeZn_13_ phase. While this method increased the tensile strength by 62%, the elongation at break remained limited to only 1.18%. Previous research by Deng et al. [15] successfully prepared a Zn-0.45Li alloy using hot extrusion and multi-pass drawing, achieving an elongation of 55.4%. Notably, rotary forging is a processing technique for bars that can significantly refine grain size and thus improve elongation [16]. For Ti [17] and Mg alloys [18], the use of rotary forging to enhance the mechanical properties has been well documented. Similarly, the rotary forging process has been applied to improve the elongation of medically degradable Zn alloys [19]. Consequently, it is anticipated that the rotary forging process can significantly enhance the plasticity of Zn-Fe alloys. Despite its advantages, rotary forging has certain limitations when applied to medical applications. For instance, rotary forging can introduce residual stresses and defects such as surface cracks [20], which may negatively affect the long-term stability of biodegradable implants [21]. Additionally, the high deformation required in rotary forging may not be suitable for all alloy compositions, as some materials may exhibit limited plasticity or increased brittleness under such conditions [22]. Hot extrusion and additive manufacturing (AM) are also widely employed techniques for fabricating zinc-based alloys, each offering unique advantages compared to rotary forging. Hot extrusion provides consistent mechanical properties along the extrusion direction and is highly scalable for industrial applications [23]. On the other hand, AM enables the production of complex and patient-specific implant geometries. However, the additive manufacturing of Zn-based alloys remains challenging due to issues such as high oxidation rates during the printing process and residual stresses in the final product [24]. Compared to these techniques, rotary forging excels in grain refinement and improving ductility, making it particularly effective for enhancing the mechanical properties of Zn-Fe alloys. Nevertheless, further optimization of the rotary forging process is necessary to address its limitations and ensure its suitability for medical applications. However, the mechanisms by which rotary forging influences the mechanical properties, degradation performance, and biocompatibility of Zn alloys are still unclear.

To investigate these issues, this study systematically examined the effects of rotary forging on Zn-0.5Fe alloys. The alloy samples were prepared through melting, casting, hot extrusion, and multi-pass rotary forging. Their microstructure was characterized using optical and scanning electron microscopy and electron backscatter diffraction (EBSD). Mechanical properties were evaluated through tensile testing, while electrochemical and immersion tests were performed to assess degradation behavior in simulated body fluid (SBF). Additionally, cytotoxicity and fluorescence staining tests were conducted to evaluate the biocompatibility of the forged samples.

## 2. Materials and Methods

### 2.1. Material Preparation

The melting, casting, and hot extrusion processes of the materials investigated in this study were carried out by Ningbo Boway Alloy Materials Co., Ltd. (Taiping Bridge, Yunlong Town, Yinzhou District, Ningbo, Zhejiang Province, 315135, China). Pure Zn (99.99 wt%) and the intermediate alloy Zn-2Fe were melted in a vacuum induction furnace under an argon atmosphere. The materials were initially placed in the induction furnace, heated to complete melting, and continuously stirred to ensure uniform mixing and slag removal. After a holding time of 30 min, the molten alloy was poured into a Ø60 mm steel mold. After cooling to room temperature, the ingots were turned and peeled to Ø50 mm. The ingots were placed in a muffle furnace and heated to a temperature of 260 °C and subjected to hot extrusion. The extrusion die was heated to 220–240 °C and held for 3 h. Asphalt was used as a lubricant, and the extrusion ratio was 20:1. Zn-0.5Fe in the extruded state was obtained by positive extrusion into an extruded bar of Ø11.2 mm.

Rods of varying diameters were produced through multi-pass rotary forging at a feed rate of 1 cm/s, based on the principle of no more than 10 % deformation in a single pass, with a total cumulative deformation of 95 %. The material undergoes deformation as a consequence of the repeated radial punching by four dies (sample diameter > 4 mm) or three dies (sample diameter < 4 mm), which rotate at high speed. Samples were taken at cumulative deformations of 90% and 95% and were designated as R1 (90%) and R2 (95%), respectively. These deformation percentages were selected to study the effects of significant grain refinement while avoiding excessive deformation that could lead to surface defects or reduced mechanical performance [25]. An initial 11 mm rod in the extruded state was used as a control with a final sample diameter of 2.45 mm.

The total deformation is calculated using the following formula:

*R*_t_ = (S_0_ − S*_n_*)/S_0_ × 100% (1)

where *R*_t_ is the total deformation, *S*_0_ is the cross-sectional area of the bar in the extruded state, and *S*_n_ is the cross-sectional area of the bar after rotary forging.

### 2.2. Microstructure Characterization

The microstructure was examined using a light microscope (AXIOLAB 5, Zeiss, Oberkochen, Germany) and a scanning electron microscope (SEM, Zeiss Sigma 300, Oberkochen, Germany). The samples intended for microscopic observation were ground on SiC abrasive paper through a series of grits, ranging from #120 to #3000, and subsequently polished by passing through a 1.5 μm diamond polisher. The samples were subjected to etching using a solution comprising chromium trioxide, sodium sulfate, and nitric acid. The orientation and antipodal maps of the alloys were investigated by electron backscatter diffraction (EBSD, Verios G4 UC, Waltham, MA, USA). The obtained EBSD data were then reconstructed using AZtecCrystal 2.1.

### 2.3. Mechanical Test

The mechanical properties were evaluated using an electronic universal testing machine (CM75105, Xi’an, China). Tensile properties were measured using 11 mm extruded specimens prepared according to the ISO 6892-1: 2019 standard [26] and machined into standard tensile specimens with a parallel length of 28.0 ± 0.1 mm and a diameter of 5 mm. To address potential instability due to the size effect, the parallel lengths of the R1 and R2 specimens were adjusted to 45 mm. Furthermore, more than three tests were performed for each group of specimens at a strain rate of 3.3 × 10^−4^ s^−1^ at room temperature. The fracture surfaces of the tensile specimens were observed using a scanning electron microscope (SEM, Zeiss Sigma 300, Oberkochen, Germany).

### 2.4. Electrochemical Measurement

Electrochemical tests were conducted using an M237A electrochemical workstation. The sample was employed as the working electrode, with a platinum plate as the counter electrode and a saturated calomel electrode (SCE) as the reference electrode. The connections were established with copper leads and sealed with epoxy resin to guarantee that the surface area of the sample exposed to SBF solution was 0.875 cm^2^. Before testing, all samples were subjected to mechanical grinding to a grit size of 3000, and the working electrode was permitted to reach equilibrium at open-circuit potential (OCP) for 20 min. Dynamic potential polarization (PDP) was tested in the range of OCP ± 0.5 V at a constant scan rate of 1 mV·s^−1^. Electrochemical impedance spectroscopy (EIS) studies were conducted at OCP with a 5 mV sinusoidal amplitude across a frequency range of 10^5^ Hz to 10^−2^ Hz. The EIS data were subjected to analysis and fitting to equivalent curves using ZSimpWin 3.60 software.

The corrosion current density (i_corr_) was determined by linear extrapolation using the Tafel method and subsequently converted to the corrosion rate (*CR*_i_, mm/year) according to the following formula [27]:

(2) $${CR}_{i}=3.27\;\times \;10^{-3}\times \frac{i_{\mathrm{corr}}}{\rho }EW,$$

where *ρ* is the density of the metal (g/cm^3^) and *EW* is the electrochemical equivalent (g).

### 2.5. In Vitro Degradation Test

The immersion test was conducted in SBF solution at a pH of 7.40 ± 0.01 and a constant temperature of 37 °C. In accordance with ASTM G31-72 [28], the ratio of the sample surface area to the volume of the SBF was determined to be 20 mL/cm^2^. The specimens were gradually sanded with 400- to 5000-grade sandpaper and polished until the scratches on the surface no longer significantly affected the corrosion process. Following immersion for a designated period, the corrosion morphology was observed using a scanning electron microscope (SEM, Zeiss Sigma 300, Oberkochen, Germany) for varying immersion times, and the composition was analyzed by energy-dispersive X-ray spectroscopy (EDS). The samples were subjected to an ultrasonic cleaning process in a 200 g/L chromium trioxide solution for five minutes to remove any corrosion products that may have formed. The corrosion rate was then calculated based on the observed weight loss. The corrosion rate was calculated by the methodology outlined in ASTM G31-72, as follows [29]:

(3) $${CR}_{w}=\frac{8.76\times 10^{4}\times ∆m}{\rho At},$$

where Δ*m* is the weight loss (g), *ρ* is the density of the material (g/cm^3^), *A* is the initial surface area of the immersed sample (cm^2^), and *t* is the immersion time (h).

### 2.6. Characterization of Cytocompatibility

Mouse fibroblast (L-929) cells (purchased from Beijing BorulKangmu Biotechnology Co., Ltd. Room 201, Building 5, Shangdi Information Industry Base, Haidian District, Beijing, 100085, China) were used following the standardized procedure outlined in ISO 10993-5:2009 [30]. The samples were subjected to an ultrasonic cleaning process with anhydrous ethanol for a duration of 10 min and sterilized under UV light for a period of 24 h. The cytocompatibility of the Zn-0.5Fe alloy samples was evaluated through the utilization of MTT colorimetry. In 96-well plates, 100 μL of cell suspension was added to each well and incubated for 24 h. The medium was replaced with different concentrations of extracts at 100%, 50%, and 25%, with normal DMEM medium without extract serving as the control. Following a 24 h incubation period, 10 μL of MTT was added to each well, and the cells were incubated for a further six hours in an incubator set at 37 °C with 5% CO_2_ and protected from light. Following the aspiration of the solution, an additional 150 μL of DMSO was added to each well, and the absorbance was measured at 550 nm using an enzyme-labeled reader (Bio-radiMark (168-1130), Hercules, CA, USA). The relative cell growth rate (RGR) was calculated according to the following formula:

(4) $$\mathrm{RGR}=\frac{\mathrm{Viable}\;\mathrm{cell}\;\mathrm{count}\;\mathrm{in}\;\mathrm{experimental}\;\mathrm{extrat}}{\mathrm{Viable}\;\mathrm{cell}\;\mathrm{count}\;\mathrm{control}\;\mathrm{extrat}},$$

L-929 cells were cultured for 24 and 72 h. Following this period, the DMEM was removed, and 40 μL of 4% paraformaldehyde was added to each well, where it was left to fix for 30 min. The paraformaldehyde was then aspirated, and 20 μL of FITC was added to each well, where it was allowed to stand for one hour. The cytoskeleton was then stained. The FITC was then aspirated, and 20 μL of DAPI was added to each well, which was then left to stand for 40 min, allowing the nuclei to be stained. The DAPI solution was then aspirated and 100 μL of PBS was added to each well. Following each aspiration, the solution in the wells was washed three times with PBS. Once the staining process was complete, the cells were fully wrapped in aluminum foil to prevent light exposure. Their morphology and number were then observed under an inverted fluorescence microscope (LEICA DMI 3000 B, Wetzlar, Germany).

## 3. Results

### 3.1. Microstructural Evolution and Mechanical Performance

**Figure 1:**
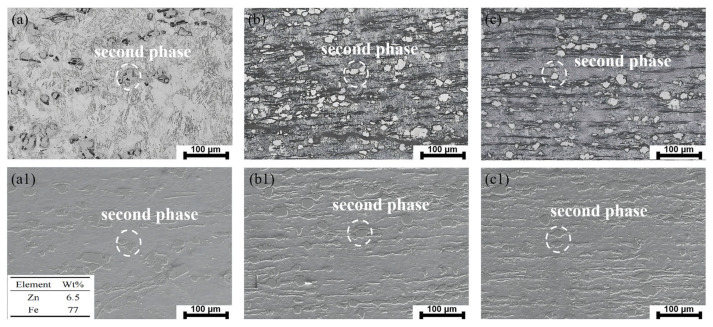
(**a**–**c**) Optical micrographs of the longitudinal sections of extruded, R1, and R2 Zn-0.5Fe alloys; (**a1**–**c1**) SEM images of longitudinal sections of extruded, R1, and R2 Zn-0.5Fe alloys.

Figure 1 shows the optical and SEM micrographs of the Zn-0.5Fe alloys. The original extruded Zn-0.5Fe alloy displays coarse grains (Figure 1a), while the grain size progressively decreases with increasing rotary forging deformation, as shown in Figure 1b,c. Rotary forging breaks the grains, leading to significant grain refinement. The EDS results for the second phase are presented in Figure 1(a1), revealing a Zn to Fe ratio close to 1:13, indicating that the second phase in Zn-0.5Fe is composed of the FeZn_13_ phase [31]. Some FeZn_13_ is disrupted by rotary forging and is more uniformly distributed than in the original extruded specimens. Although the grain size of the zinc matrix is significantly refined by rotary forging, the FeZn_13_ phase size remained unchanged, with only a slight reduction.

**Figure 2:**
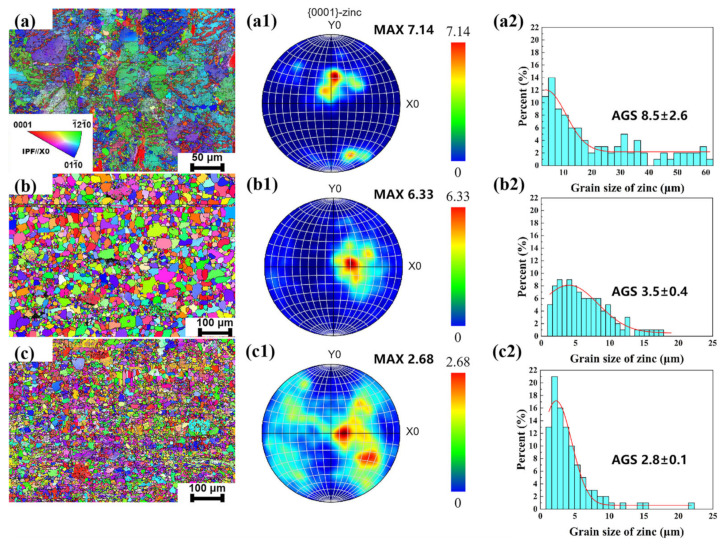
EBSD orientation diagram, pole diagram, and inverse pole diagram of Zn-0.5Fe alloy: (**a**–**a2**) extruded, (**b**–**b2**) R1, and (**c**–**c2**) R2.

Figure 2 shows the EBSD results for the Zn-0.5Fe alloy. The inverse polar figure (IPF) of the extruded Zn-0.5Fe alloy (Figure 2a) reveals coarse grains oriented along the (−12–10) and (01–10) directions. Figure 2b,c show the IPF of the R1 and R2 samples, respectively. The grain size decreased with rotary forging deformation, leading to the formation of equiaxed grains. Furthermore, energy is dissipated in the form of heat during severe plastic deformation of the material, resulting in a special thermal effect [32]. The synergistic effect of thermal effects and rotary forging deformation is to rotate and reorganize the grains during the deformation process while new grain orientations are formed. Therefore, the grain orientation along (0001) increased, while the orientations along (−12–10) and (01–10) decreased after deformation.

The textures of the Zn-0.5Fe alloy were analyzed using polar figure (PF), as shown in Figure 2(a1–c1). The texture of the extruded Zn-0.5Fe alloy mainly exhibits the (0001)//Y0 orientation, with a multiple uniform density (MUD) value of 7.14. After rotary forging, the texture shifts to (0001)//X0, and the texture strength decreases as deformation increases, with MUD values reducing to 6.33 and 2.68 for R1 and R2, respectively. Two primary factors lead to this change: (i) The extruded Zn-0.5Fe alloy has large, inhomogeneous grains that concentrate grain orientation. Rotary forging refines grain size, improves inhomogeneity, and reduces selective orientation. (ii) The dynamic recrystallization process of the material is stimulated by severe plastic deformation and additional thermal effects, resulting in continuous dynamic recrystallization occurring at room temperature [32,33,34]. On the other hand, the FeZn_13_ phase in rotary forging hinders grain boundary migration, delaying grain growth. Figure 2(a2–c2) show that the average grain size of extruded samples is 8.5 µm, while the grain size of R1 and R2 is reduced to 3.5 µm and 2.8 µm, respectively.

**Figure 3:**
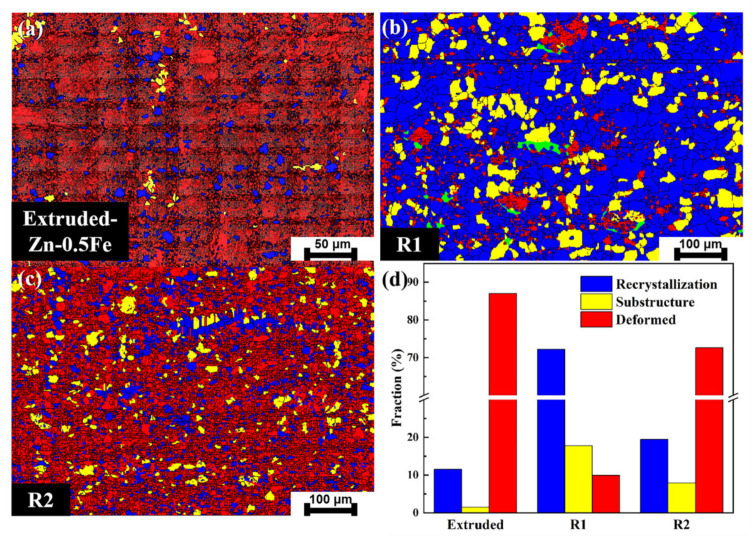
Distribution of recrystallized grains: (**a**) extruded, (**b**) R1, (**c**) and R2. (**d**) Recrystallization fraction statistics of Zn-0.5Fe alloy.

Figure 3 shows the distribution of recrystallized grains. The blue, yellow, and red areas are recrystallized grains, substructure grains, and deformed grains, respectively. Compared with the extruded sample, the recrystallized grains occupy 72.2% and 19.5% in R1 and R2, respectively. The percentage of recrystallized grains is higher in both samples than in the initial extruded sample (Figure 3d). This suggests that deformation and temperature effects during the forging process cause dynamic recrystallization. The substructure and recrystallized grains were reduced from R1 to R2. This phenomenon can be attributed to the concurrent acceleration of nucleation growth and the progression of dynamic recrystallization, which leads to a decrease in the proportion of recrystallized grains.

**Figure 4:**
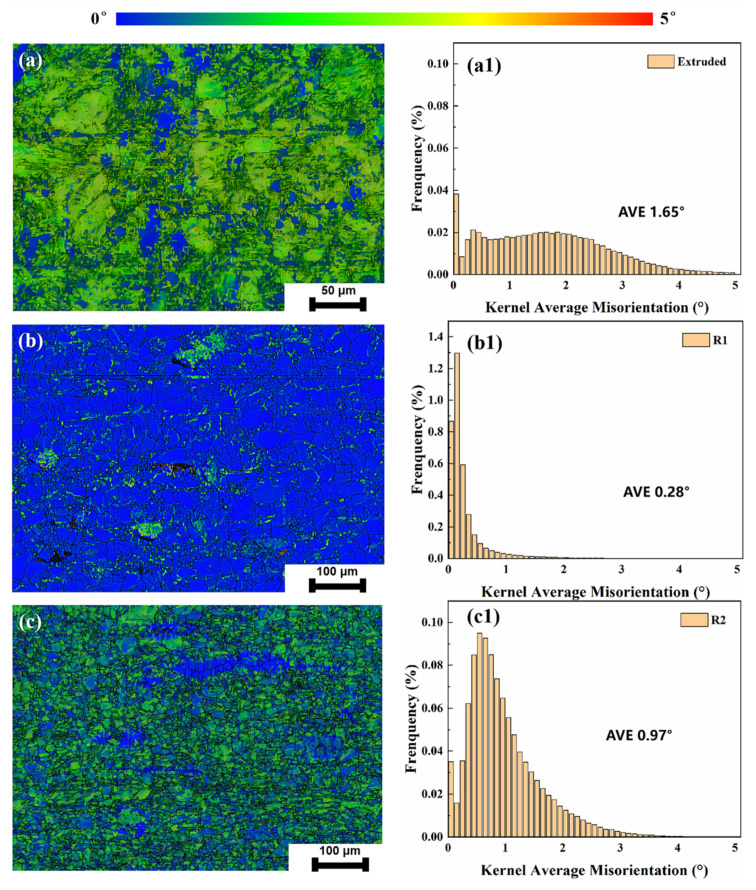
KAM maps of the Zn-0.5Fe alloy: (**a**,**a1**) extruded, (**b**,**b1**) R1, and (**c**,**c1**) R2.

Figure 4 shows the kernel average misorientation (KAM) results of extruded, R1, and R2 samples, reflecting the variation in dislocation density of each state. As shown in Figure 4(a1–c1), the KAM values decrease from 1.65 for the extruded sample to 0.28 for R1 and 0.97 for R2. This indicates that dynamic recrystallization, induced by rotary forging effectively reduces dislocation density during the deformation process.

**Figure 5:**
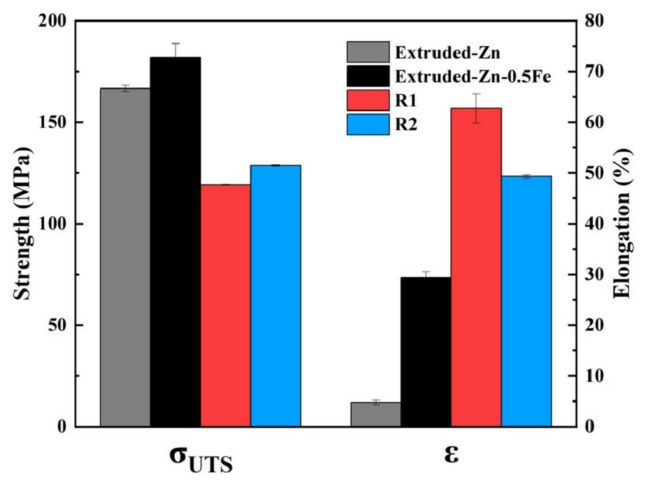
Tensile strength and elongation of Zn-0.5Fe alloys at room temperature.

**Figure 6:**
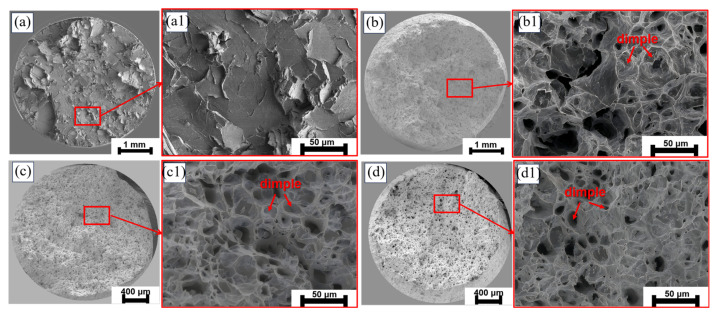
Fracture morphology: (**a**,**a1**) extruded Zn, (**b**,**b1**) extruded Zn-0.5Fe, (**c**,**c1**) R1, and (**d**,**d1**) R2.

Figure 5 shows the tensile strength and elongation of pure Zn and the Zn-0.5Fe alloy at room temperature. Alloying leads to a moderate increase in tensile strength and a significant improvement in ductility, with the Zn-0.5Fe alloy achieving 182 MPa and 28%, respectively. Nevertheless, 60% and 50% elongations were attained in the R1 and R2 states, with 114% and 79% increases, respectively, compared to the extruded Zn-0.5Fe alloy. Figure 6a,d show the fracture morphology of pure Zn and Zn-0.5Fe alloys. Figure 6a shows minimal necking of the extruded pure Zn. A further enlarged fracture of the pure Zn exhibited brittle fracture and was tearing, as illustrated in Figure 6(a1). Figure 6b illustrates ductile fracture characteristics in the extruded Zn-0.5Fe. Figure 6c,d represent the tensile fracture morphologies of R1 and R2. The formation of dimples is more pronounced, and the depth of these dimples increases following rotary forging.

Dynamic recrystallization and recovery occur during the rotary forging process. The dynamic recovery mechanism removes stress concentrations by rearranging dislocations, enabling partial structural recovery of the material during deformation. This process reduces the dislocation–strengthening effect as dislocations are continuously annihilated, leading to a reduction in the strength of Zn-0.5Fe alloys after rotary forging. However, the elongation of the Zn-0.5Fe alloy increased due to grain refinement and a higher proportion of grain boundaries. For the R1 sample, elongation improved to 60%, while its UTS reached 119 MPa. However, the elongation in R2 did not increase but rather decreased compared to R1, indicating that further grain refinement could not increase the elongation of the Zn-0.5Fe alloy. This phenomenon may be associated with the brittle phase FeZn_13_. During the forging process, the formation of the complex and brittle FeZn_13_ phase results in work hardening within the Zn-0.5Fe alloy, rendering it more susceptible to stress concentrations. The FeZn_13_ phase acts as a barrier to grain boundary migration, which contributes to grain refinement; however, its brittleness can create localized stress concentrations during deformation, particularly under high-deformation conditions such as in R2. These stress concentrations increase the likelihood of microcracks forming and propagating, thereby reducing the elongation of the alloy despite further grain refinement. This balance between grain refinement and stress concentration underlines the importance of optimizing the volume fraction and distribution of FeZn_13_ phases to balance strength and ductility in Zn-Fe alloys. As demonstrated in Figure 4, it is evident that the dislocation density in the R2 sample exhibits an increase in comparison with R1. This indicates an enhancement in the material’s sensitivity to stress concentration, leading to a reduction in the elongation of the material. To address this issue, several potential mechanisms can be employed to mitigate the brittle behavior caused by FeZn_13_ phases: (i) heat treatment (annealing can be used to reduce residual stress and stabilize the phase structure, thereby improving the ductility of the alloy), (ii) optimization of rotary forging parameters (adjusting the forging process, such as increasing the deformation rate or temperature, can facilitate the breaking of FeZn_13_ phases into smaller fragments, reducing their size and mitigating their brittle nature), and (iii) refinement during casting (employing rapid cooling techniques during alloy melting and casting can lead to the formation of smaller and more uniformly distributed FeZn_13_ phases, which would decrease the stress concentration during deformation).

These strategies provide a pathway to balance the strength and ductility of Zn-Fe alloys, further enhancing their suitability for biomedical applications. However, further experimental studies are needed to evaluate the effectiveness of these approaches in mitigating the brittle behavior of FeZn_13_ phases.

A further reduction in grain size is observed from R1 to R2. However, the proportion of recrystallized grains decreases during this process, indicating that the mechanism of strength reduction due to dynamic recrystallization is no longer dominant. At this stage, the Hall–Petch effect within the material is evident, resulting in enhanced strength of R2 compared to R1. Furthermore, the R2 specimens exhibit a size effect, and the accumulated processing passes lead to defects that ultimately destabilize the tensile process [35]. Accordingly, the elongation of R2 decreases, and the UTS increases compared to R1.

### 3.2. Characterization of Degradation Properties

**Figure 7:**
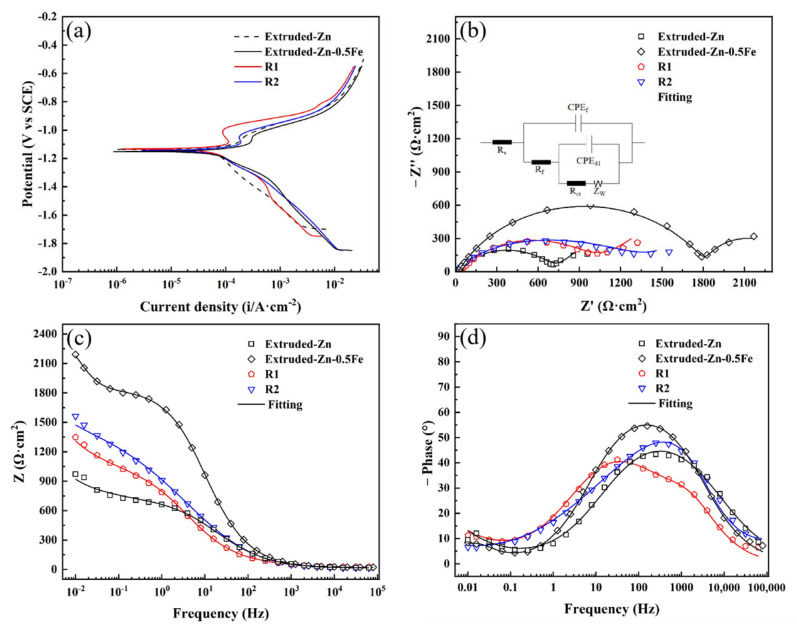
Polarization curves and EIS results of different Zn alloys in SBF solution: (**a**) polarization curve, (**b**) Nyquist plot, and (**c**,**d**) Bode plots.

Impedance is an effective method to investigate the electrochemical corrosion process of metals. The EIS curves and the corresponding dynamic potential polarization obtained by immersing the specimens in SBF are illustrated in Figure 7. It can be observed that the corrosion potential of the Zn-0.5Fe alloy is not sensitive to the amount of deformation of the material. The EIS response of the specimens exhibits two semi-circular curves. Nyquist plots indicate that the diameter of the capacitive arc of the Zn-0.5Fe alloy in the extruded state increases with the addition of elemental Fe, thereby suggesting that elemental Fe improves the corrosion resistance of pure Zn [36]. Furthermore, the kinetic potential polarization curves matched with the EIS also indicate that the corrosion resistance of the Zn-0.5Fe alloy in the extruded state is superior to that of pure Zn.

The corrosion resistance of R1 and R2 samples was observed to decline with grain refinement induced by the rotary forging deformation. This finding is supported by the polarization curves analyzed using the Tafel extrapolation method and are presented in Table 1. However, the corrosion resistance of R2 has been observed to recover in comparison to R1. This is attributed to the finer grain of R2, which results in a more uniform passivation layer and a more protective passivation film. The polarization curves of the Zn-0.5Fe alloy exhibit a distinct passivation zone in the anodic region, which suggests that the SBF environment is conducive to the alloy’s passive protection [37].

The equivalent circuit of the Zn-0.5Fe alloy immersed in SBF solution, fitted based on the EIS diagram, is illustrated in Figure 7b. In this diagram, Rs represents the solution resistance between the reference electrode and the working electrode, while Rf denotes the film resistance at the surface. A constant phase element (CPE) is a device used to compensate for inhomogeneities in a system, such as surface inhomogeneities [38]. The Warburg impedance (w) is a measure of the rate of diffusion of ions in a solution [39]. The fitted parameters are presented in Table 2, and the chi-squared value (x^2^) is less than 1.13 × 10^−3^, indicating an excellent fit to the experimental data.

**Figure 8:**
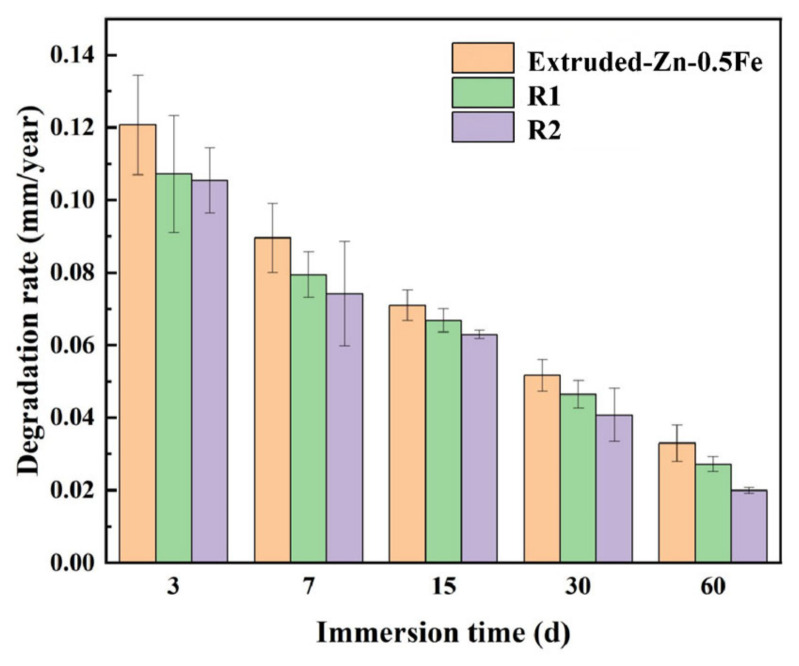
Corrosion rate of Zn-0.5Fe with different diameters after being soaked in SBF for different numbers of days.

Figure 8 illustrates the degradation rate of the Zn-0.5Fe alloy, determined through weight loss measurements after immersion in SBF for varying durations. The results indicate a monotonically decreasing degradation rate for all samples over time. This is attributed to the progressive accumulation of corrosion products on the alloy surface during the initial stages. These corrosion products form a protective layer, impeding further degradation by limiting the exposure of the underlying material to the corrosive environment. The extruded Zn-0.5Fe alloy exhibited the highest degradation rate of 0.12 mm/year within the first three days of immersion, remaining well below the 0.2 mm/year standard degradation rate required for biodegradable implantable devices [40]. This performance highlights its suitability as a medical-grade material for controlled biodegradation. The degradation rates of the rotary-forged samples (R1 and R2) also fell within this clinically acceptable range. For the rotary-forged samples, a similar trend of decreasing degradation rates was observed, although with notable variations. As the degree of deformation increased from the extruded state to R1 and R2, the grain refinement induced by rotary forging enhanced the compactness and uniformity of the corrosion product layer. The R2 samples, which experienced the highest deformation, displayed a particularly dense passivation layer that further reduced the degradation rate compared to R1. In practical clinical applications, the degradation rate of biodegradable implants should balance mechanical stability and timely bio-resorption to facilitate tissue healing. The observed trends suggest that the Zn-0.5Fe alloy, particularly after rotary forging, can achieve this balance by providing initial mechanical support while degrading at a controlled rate suitable for orthopedic applications. However, further in vivo studies are needed to confirm whether these in vitro degradation rates align with the specific requirements of different implantation sites and conditions. This improved corrosion resistance can be attributed to the refined microstructure and increased grain boundary area, which facilitate the formation of a stable and cohesive protective layer. Consequently, at each immersion time point, the corrosion rate decreased progressively from the extruded state to R1 and R2, underscoring the effectiveness of the rotary forging in enhancing the degradation behavior of Zn-0.5Fe alloys. These findings validate the potential of Zn-0.5Fe alloys as biodegradable implant materials, with rotary forging offering a promising approach to optimizing their corrosion performance and ensuring controlled in vivo degradation.

**Figure 9:**
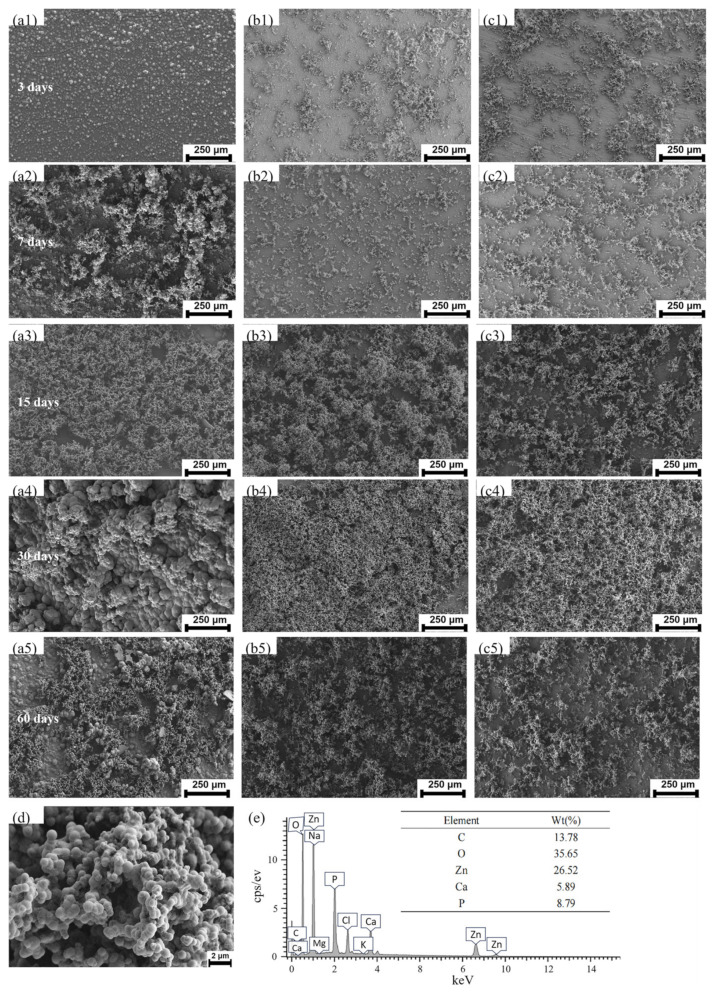
Surface morphology of Zn-0.5Fe with different diameters soaked in SBF for different numbers of days under the SEM: (**a1**–**a5**) extruded; (**b1**–**b5**) R1; (**c1**–**c5**) R2; (**d**,**e**) EDS of corrosion products.

Figure 9 presents the EDS analysis and SEM images of the corrosion morphology and products formed on Zn-0.5Fe samples immersed in SBF for 3, 7, 15, 30, and 60 days. The results reveal that the corrosion products initially exhibit a granular distribution, with some clusters of particles sparsely scattered across the surface. During the early stages of immersion (e.g., 3 days), a significant portion of the sample surface remains exposed, indicating incomplete coverage by corrosion products. As the immersion period increases, these granular corrosion products gradually amalgamate into larger clusters, forming a more uniform and continuous layer across the sample surface. By the 15th day, the aggregates become denser and begin to form island-like structures, which increasingly cover the sample surface. After 30 and 60 days of immersion, a nearly complete and dense layer of corrosion products is observed.

However, for smaller-diameter samples subjected to higher degrees of rotary forging deformation, the increased surface curvature accelerates localized detachment of the corrosion layer at later stages. This phenomenon leads to the formation of alternating regions of exposed surfaces and clusters of corrosion product. The degree of rotary forging deformation significantly affects the distribution and density of the corrosion product layer. For instance, samples with higher deformation levels, such as R2, show a more stable and dense coverage of corrosion products due to enhanced grain refinement and uniformity in microstructure. These microstructural changes promote a more cohesive passivation layer, reducing susceptibility to localized corrosion. EDS analysis (Figure 9e) identifies the primary constituents of the corrosion products as carbon, oxygen, zinc, calcium, and phosphorus, suggesting the formation of zinc oxides, carbonates, and phosphates during immersion. These compounds play a critical role in forming a stable corrosion product layer that mitigates further material degradation.

### 3.3. Cytotoxicity Test and Fluorescence Staining

**Figure 10:**
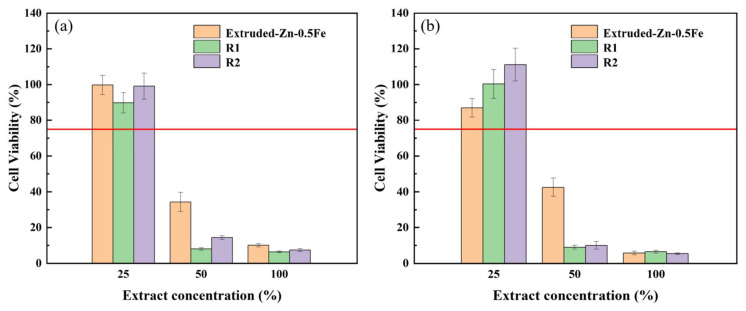
Cell viability of L-929 cultured in 25%, 50%, and 100% Zn-0.5Fe alloy extracts for (**a**) 1 day and (**b**) 3 days. The red line in the figure represents the 75% RGR value, which is the threshold for determining the presence or absence of cytotoxicity.

Figure 10 presents the results of the cytotoxicity assay, evaluating the RGR of L-929 cells after 1 and 3 days of incubation with different concentrations of Zn-0.5Fe alloy extracts. The extracts were tested at 100%, 50%, and 25% concentrations, with a standard DMEM medium serving as the control group. The RGR values for all samples remained within the acceptable threshold for cytocompatibility according to ISO 10993-5, which defines an RGR above 75% as non-cytotoxic. The results for the 50% and 100% extracts indicate a concentration-dependent reduction in cell viability. At higher extract concentrations, the relative growth rate (RGR) fell below 75%, which does not meet the ISO 10993-5 standard for cytocompatibility. This suggests that the higher ion concentrations released from the alloy may negatively affect cell metabolism or viability. However, the results at lower concentrations (e.g., 25% extract) remained within acceptable cytocompatibility limits. After 1 day of incubation, among the samples, R2 exhibited the most favorable cytocompatibility, suggesting that the microstructural alterations induced by large deformation enhance the material’s cytocompatibility. This outcome aligns with the corrosion rate pattern observed in the in vitro immersion test, with the relative growth rate values consistently within the 75% range at 25% leachate concentration. After 3 days of incubation, all samples exhibited RGR values still exceeding 75% at 25% extract concentration, indicating a strong ability to support cell viability and proliferation. This cytocompatibility is attributed to the microstructural refinement induced by rotary forging, which creates a more homogeneous alloy matrix.

**Figure 11:**
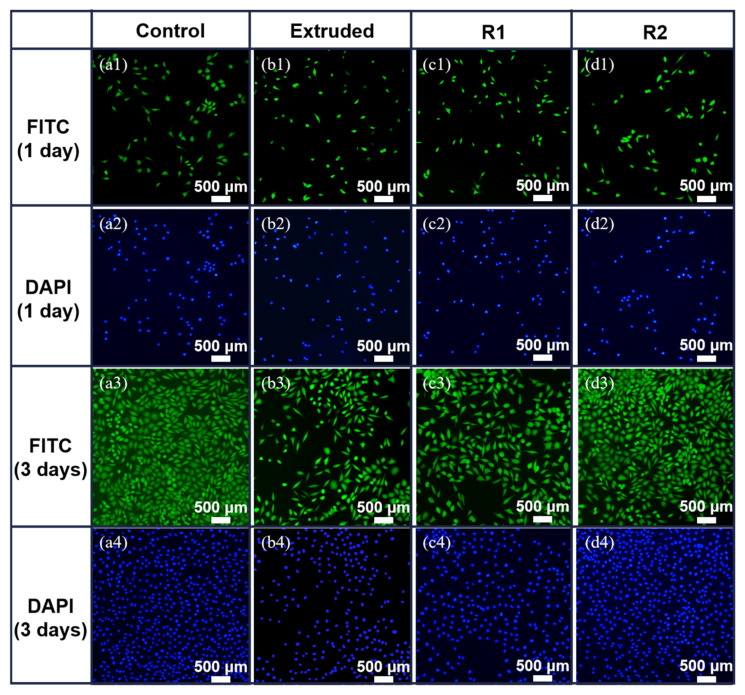
Fluorescence staining of L-929 cultured in 25% Zn-0.5Fe alloy extract for 1 day and 3 days: (**a1**–**a4**) Control; (**b1**–**b4**) Extruded; (**c1**–**c4**) R1; (**d1**–**d4**) R2.

Figure 11 illustrates the cell morphology observed through fluorescence staining of the cytoskeleton and nucleus using FITC (green) and DAPI (blue). This provides a complementary visualization of the cytotoxicity assay results shown in Figure 10. On the first day of incubation, the number of L-929 cells was relatively low, and their morphology exhibited a mixture of rounded and spindle-shaped cells. Despite the morphological differences, the nuclei in all groups remained intact, suggesting no significant cytotoxic effects from the Zn-0.5Fe alloy extracts. By the third day, the cell density increased significantly across all groups, with a noticeable rise in the percentage of spindle-shaped cells, indicative of enhanced cell adhesion and spreading. The R2 sample, which had the highest RGR as shown in Figure 10, exhibited the most pronounced improvements in cell morphology, characterized by well-spread cytoskeletons and intact nuclei. This improvement aligns with the superior cytocompatibility of the R2 alloy.

## 4. Conclusions

This study investigated the effects of rotary forging on the mechanical, degradation, and biocompatibility properties of Zn-0.5Fe alloys. The following conclusions were drawn:

(1) Microstructural refinement: Rotary forging significantly refined the grain structure and optimized the texture distribution of Zn-0.5Fe alloys. The dynamic recrystallization and deformation-induced thermal effects contributed to grain refinement and improved homogeneity.
(2) Mechanical properties: The elongation of the Zn-0.5Fe alloy increased by 114% after rotary forging, reaching 60%. The process facilitated the formation of fine-grain structures, indicating improved ductility and toughness.
(3) Degradation behavior: Electrochemical and immersion tests demonstrated that rotary forging enhances the formation of a dense and uniform corrosion product layer. The Zn-0.5Fe alloy exhibited a stable degradation rate within acceptable limits for biodegradable implants.
(4) Biocompatibility: Cytotoxicity tests and fluorescence staining revealed excellent biocompatibility of the Zn-0.5Fe alloy after rotary forging, with favorable cell viability, especially in samples subjected to higher deformation levels.

In summary, rotary forging effectively improves the mechanical, corrosion, and biological performance of Zn-0.5Fe alloys. This study also has certain limitations. Exploring the effects of additional alloying elements and optimizing rotary forging parameters (e.g., strain rates and deformation levels) could further enhance the alloy’s properties and broaden its potential clinical applications. Furthermore, all tests were conducted under in vitro conditions, which may not fully replicate the complexities of in vivo environments, particularly in terms of long-term interactions with biological tissues. Additionally, the influence of dynamic physiological conditions on the alloy’s mechanical integrity and degradation behavior has not been addressed.

## Figures and Tables

**Table 1:** Polarization curve fitting results obtained for the corrosion rate of Zn-0.5Fe alloy.

Alloy	E_corr_ (v)	i_corr_ (μA/cm^2^)	Corrosion Rate (mm/Year)
Extruded Zn	−1.14	34.97	0.41
Extruded Zn-0.5Fe	−1.13	53.03	0.62
R1	−1.14	34.72	0.40
R2	−1.15	48.07	0.56

**Table 2:** Fitting parameters of Zn-0.5Fe alloy in SBF.

Alloy	*R_s_* (Ω·cm^2^)	*R_f_* (Ω·cm^2^)	*R_cf_* (Ω·cm^2^)	χ^−2^ (×10^−4^)
Extruded Zn	13.93	710.6	2.83 × 10^7^	9.43
Extruded Zn-0.5Fe	21.21	1828	518.5	4.32
R1	26.46	60.25	952.3	8.01
R2	8.18	13.81	1468	7.11

## Data Availability

The original contributions presented in the study are included in the article; further inquiries can be directed to the corresponding author.
